# Case Report: Arthroscopic treatment of calcific periarthritis of the knee in the lateral and medial collateral ligaments

**DOI:** 10.3389/fsurg.2025.1466031

**Published:** 2025-07-14

**Authors:** Changli Liu, Guosheng Yu, Xiuquan Zhao, Yanzhao Hu

**Affiliations:** ^1^ Department of Orthopaedic Surgery, Cangzhou Hospital of Integrated Traditional Chinese and Western Medicine of Hebei Province, Cangzhou, Hebei, China; ^2^ Hebei Key Laboratory of Integrated Traditional and Western Medicine in Osteoarthrosis Research (Preparing), Cangzhou, Hebei, China

**Keywords:** case report, pathologic calcification, medial collateral ligament (MCL), lateral collateral ligament (LCL), arthroscopy

## Abstract

**Objective:**

Calcific periarthritis, a well-recognized pathological condition of the shoulder joint, also represents an uncommon etiology of severe knee pain. Here, we present a case report of a patient with calcifications concurrently affecting both the lateral collateral ligament (LCL) and medial collateral ligament (MCL).

**Case description:**

The 62-year-old woman presented with severe lateral knee pain and restricted range of motion in her right knee. The knee exhibited marked tenderness in the posterolateral region and slight swelling, and maintained ligamentous stability. X-ray imaging of the knee revealed well-defined calcific deposits bilaterally on the femoral condyles. MRI findings indicated hypointense signal areas in close proximity to the insertions of the LCL and MCL. Laboratory test results were within normal limits. A preliminary diagnosis of calcific periarthritis was established based on comprehensive clinical assessments. Given the ineffectiveness of conservative interventions and at the patient's strong request, arthroscopic surgery was performed specifically targeting the calcific deposition in the LCL.

**Results:**

The patient experienced immediate relief of symptoms following the operation. Intraoperative biopsy validated the initial diagnosis. During the 2-year follow-up period, the patient remained pain-free, and radiographic assessment indicated asymptomatic dissolution of the calcific deposition in the MCL.

**Conclusion:**

This study represents the first documentation of calcifications affecting both the LCL and MCL simultaneously. The progression of MCL calcifications demonstrated the potential for asymptomatic presentation from onset to resolution. In cases where conservative management fails to address symptomatic calcific disease of the LCL, arthroscopic surgery may be warranted.

## Introduction

Calcific periarthritis, like calcific tendinitis, is part of the disease spectrum of hydroxyapatite deposition disease. The disease is an inflammatory condition characterized by the aberrant deposition of hydroxyapatite crystals within the tendon, ligament or capsule in a periarticular location, leading to intense pain and restricted joint mobility. Although the rotator cuff of the shoulder, particularly the supraspinatus tendon, is the most commonly affected site, isolated cases have been reported in various other locations such as the hip, elbow, wrist, knee, and other joints (1). Although calcific deposits around the knee is considered rarely, sporadic reports exist in the literatures (2, 3). To our knowledge, the coexistence of calcific periarthritis in the lateral collateral ligament (LCL) and medial collateral ligament (MCL) has not been previously documented. Conservative treatment is typically effective. However, when conservative measures prove inadequate, invasive intervention, such as ultrasound-guided percutaneous irrigation and arthroscopy, can offer definitive pain relief and aid in early-phase rehabilitation (4).

Here, we present a case involving calcific periarthritis of the LCL accompanied by calcific deposits in the MCL. Following unsuccessful non-surgical management, significant pain relief was achieved with arthroscopic surgery. The diagnosis was confirmed through subsequent pathological examination.

## Case description

A 62-year-old woman presented with acute onset of right knee pain and restricted range of motion while hospitalized for hypertension. She reported inability to bear full weight on the affected knee and denied any history of prior pain, trauma, sepsis, or gout in the joint. There was no significant family history of similar conditions. Physical examination revealed pronounced tenderness on the posterolateral aspect of the knee, mild swelling, and an absence of erythema, effusion, or warmth. No pain was noted in other areas of the joint, and ligament stability was confirmed. Active and passive range of motion was limited to 0°–90°, with further flexion eliciting significant pain. The patient declined further examination owing to severe pain.

**Figure 1:**
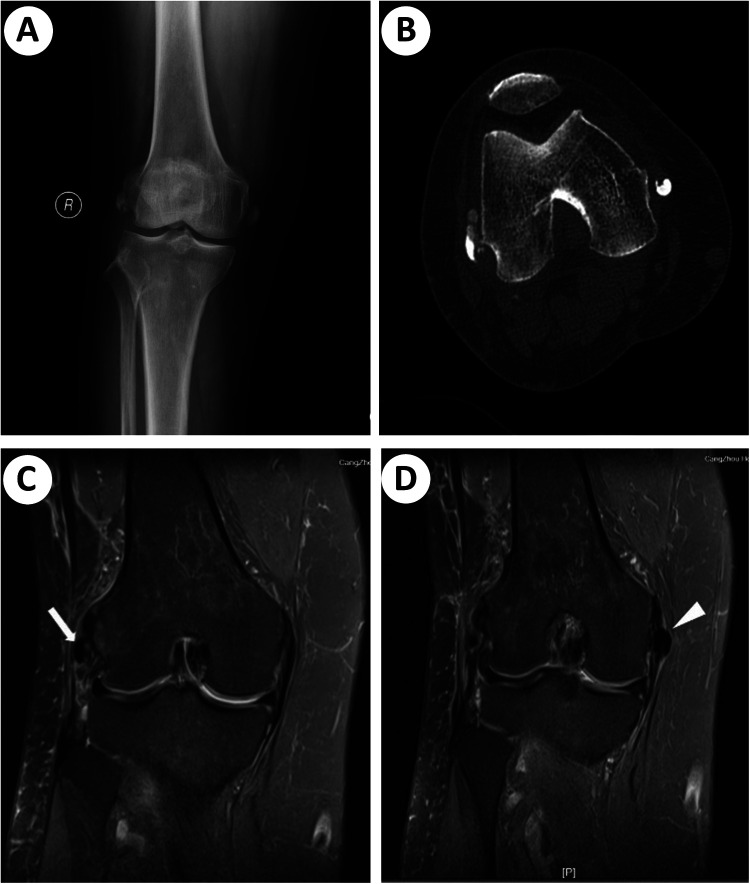
Preoperative radiographic findings. **(A)** X-ray showing well-defined calcification deposits in close proximity to the bilateral femoral condyles. **(B)** Axial CT image displaying calcifications. **(C)** Coronal MRI views of the affected LCL. Zone of hypointense signal (white arrow) suggesting calcific deposit. **(D)** Coronal MRI views of the affected MCL. Calcification (white arrowhead) adjacent to the proximal insertion of the MCL.

Anteroposterior radiography and computed tomography (CT) imaging of the knee revealed well-defined calcific deposits on both femoral condyles (Figures 1A,B). Magnetic resonance imaging (MRI) indicated hypointense signal regions near the insertions of the lateral collateral ligament (LCL) and medial collateral ligament (MCL), with surrounding edematous changes observed around the LCL (Figures 1C,D). No signs of avulsion injury or fracture were noted. Laboratory investigations, including erythrocyte sedimentation rate, C-reactive protein levels, and routine blood tests, yielded normal results. The patient also underwent rheumatological screening (including serum uric acid and anti-CCP antibodies), and no evidence of systemic rheumatic diseases was identified. A provisional diagnosis of calcific periarthritis was established based on the overall clinical findings.

Conservative treatment involved ice therapy, immobilization with splints, and oral non-steroidal anti-inflammatory drugs (NSAIDs) for a 2-week period. Despite these efforts, the severe symptoms were not adequately relieved. Due to severe pain and limited mobility, the patient had lost confidence in conservative treatment and therefore strongly requested surgical intervention. Because the calcific deposition in the MCL was asymptomatic and located extracapsularly—necessitating an additional incision for excision—a decision was made, following discussion with the patient, to address the calcific deposition solely in the LCL.

**Figure 2:**
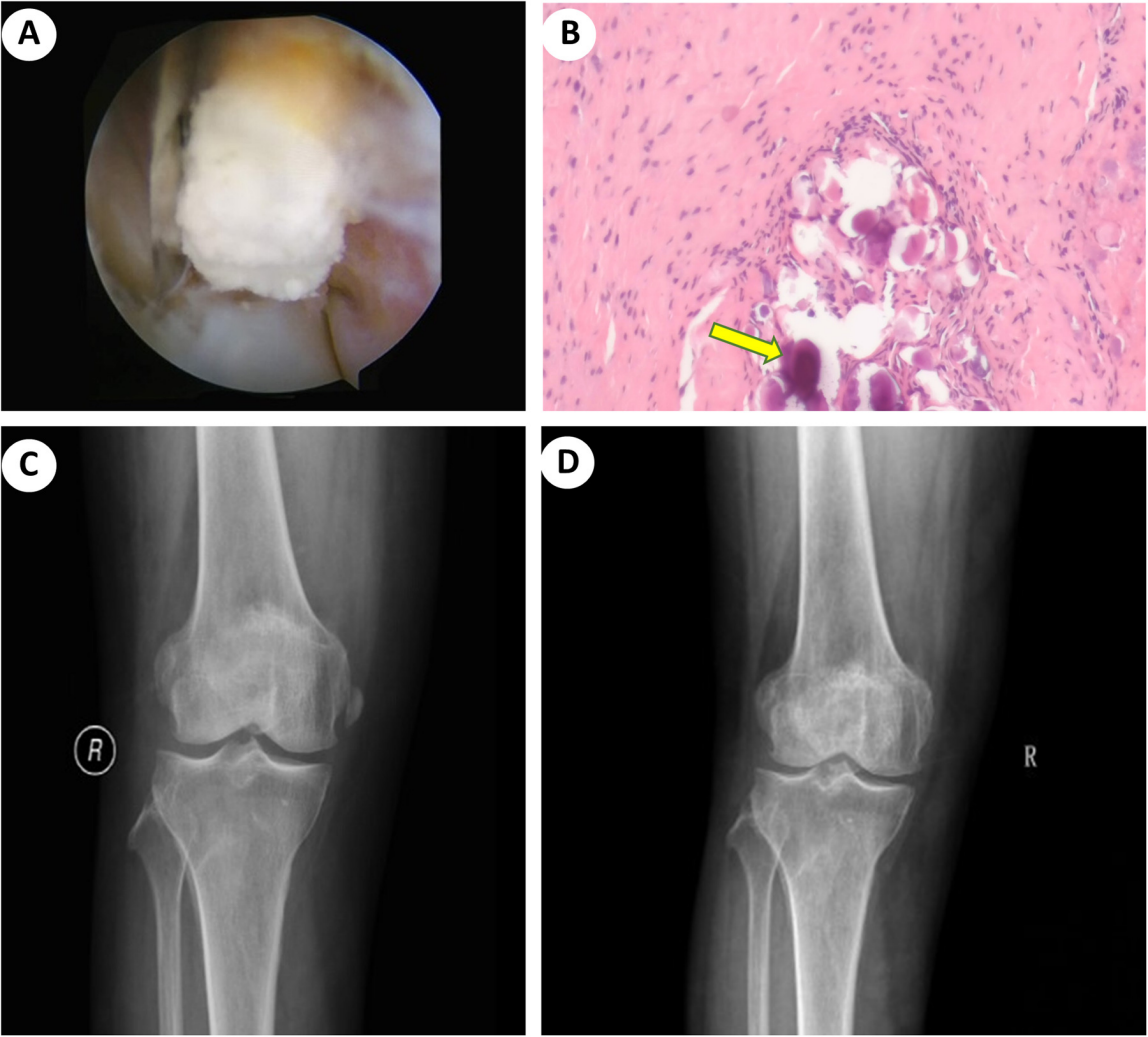
Intraoperative and postoperative images. **(A)** Arthroscopic technique demonstrating the removal of calcification from the LCL using a shaver. **(B)** Histopathology showed inflammation of the synovial tissue with calcific deposition (yellow arrow). **(C)** Postoperative x-ray demonstrating complete debridement of calcific deposits from the LCL. **(D)** AP radiograph indicating no signs of recurrence, with resolution of calcification in the MCL, which had dissolved at 2-years follow-up.

The patient was positioned supine following subarachnoid anesthesia. Arthroscopic evaluation revealed a degenerative knee joint with notable synovial proliferation in the lateral gutter. With the knee extended, the arthroscope was inserted through the anterolateral portal for visualization, and a shaver was introduced through the superolateral portal to decongest the synovium. In the lateral sulcus, without excessive disruption of the joint capsule, A deposit nodule can be identified between the LCL and the popliteus tendon (Figure 2A). Following creation of a rent using the shaver, a toothpaste-like material that extruded from the deposit was obtained for biopsy. Subsequently, complete debridement and irrigation of the deposit were performed, resulting in minimal ligament damage from the debridement process. A partial synovectomy was then carried out.

Postoperatively, the patient experienced rapid relief of symptoms, enabling near full weight-bearing ambulation. Histopathological examination revealed synovial cell hyperplasia, thickening of the subsynovial layer, inflammatory cell infiltration, proliferation of small blood vessels, and scattered necrotic foci. Calcium salt deposition was also observed, consistent with the diagnosis of calcific periarthritis (Figure 2B). Subsequent x-ray imaging confirmed complete removal of the calcific deposit from the LCL (Figure 2C). During the 2-month follow-up, the patient remained pain-free and was able to resume nearly all routine activities comfortably. Furthermore, clinical and radiographic evaluations over the subsequent 2 years demonstrated no signs of recurrence, with complete dissolution of calcification in the MCL and no associated clinical symptoms (Figure 2D).

## Discussion

Calcific periarthritis typically manifests in middle-aged and older individuals, with a slightly higher incidence rate observed in women than men. It occurs in approximately 3% of adults, with the rotator cuff tendons being the most frequently affected site (5). In over 75% of cases, the supraspinatus tendon is involved (6).

Following the rotator cuff, the most commonly affected sites, in order of frequency, are the spine, foot, and ankle. Involvement of the knee joint is relatively rare, accounting for approximately 2.5% of non-shoulder calcific periarthritis cases (7). Calcific periarthritis is considered an uncommon etiology of knee pain. Previous reports have seldom described involvement of LCL or MCL. The LCL, an extra-articular structure, is recognized as one of the four major ligaments of the knee joint. Proximally, it originates from the posterior to the bony ridge of the lateral femoral epicondyle. Distally, it inserts onto a superior and laterally facing V-shaped plateau on the fibular head. The ligament exhibits an elliptical or cord-like cross-sectional profile with a mean length of 66 mm. It serves as the primary stabilizer against varus forces and is also recognized to restrict external rotation during knee flexion. As the knee flexes, the LCL slackens (8, 9). To date, there have been no documented cases of calcific periarthritis involving the LCL coexisting with calcific deposition in the MCL. In Kamawal's study, a patient was documented to have calcific lesions in the MCL and rotator cuff, however, these did not occur simultaneously, but in a sequential manner (10).

The precise etiology and pathogenesis of calcific periarthritis or tendinopathy remain a subject of ongoing debate. Calcific tendinitis is characterized as an inflammatory process mediated by cellular mechanisms (11). Calcification typically presents in a multifocal pattern and is subsequently resorbed through spontaneous phagocytosis. Calcific tendinitis can manifest as a primary condition or as a secondary phenomenon associated with various diseases, including end-stage renal failure, tumoral calcinosis, collagen vascular disorders, diabetes mellitus, and vitamin D toxicity (12). Endocrine disorders, particularly imbalances in estrogen and thyroxine, are known to increase susceptibility to this condition. The accumulation of glycosaminoglycans in the extracellular matrix because of hypothyroidism can contribute to tendon calcification (13). Initially, calcific tendinitis was thought to result from tendon tissue degeneration, a theory widely accepted at the time. However, subsequent studies have suggested that the etiology of calcific tendinitis may involve tendon cell necrosis induced by hypoxia, leading to intracellular calcium accumulation (14). Avascularity is considered a key factor contributing to decreased local oxygen tension and subsequent hypoxia. It is important to note that calcific tendinitis differs from degenerative calcification both histologically and at the ultrastructural level (15).

Based on its presentation and pathoanatomy, the progression of calcific periarthritis or tendinopathy can be delineated into three distinct stages: the pre-calcific stage, calcific stage, and post-calcific stage. Furthermore, the calcific stage is further subdivided into the formative phase, resting phase, and resorptive phase (15). The characteristics of the deposition change from a chalk-like consistency to a thicker, creamy, or toothpaste-like material during the calcific stage. Although the majority of the calcification process is asymptomatic, pain tends to become severe as the calcific deposition begins to undergo resorption during the inflammatory process (16). Recent research has emphasized the role of neovascularization and nerve ingrowth in the manifestation of symptoms (11).

In the present case, calcific periarthritis of the LCL presented with the sudden onset of acute knee pain, consistent with the typical manifestations of calcific periarthritis. Affected knees exhibit significant tenderness and limited range of motion, and may display warmth, erythema, or effusion. Patients may experience difficulty bearing full weight on the affected side. There is usually no history of prior trauma. Erythrocyte sedimentation rate and C-reactive protein levels are typically within normal limits or slightly elevated. Patients with calcific periarthritis do not typically show abnormalities in blood calcium and phosphorus levels (17). The initial diagnosis of a “locked knee” may be considered due to knee pain and muscle spasm leading to restricted movement (18). Acute and severe knee joint pain associated with functional limitations, in the absence of a history of injury, should raise suspicion of calcific periarthritis. The clinical presentation can mimic that of gout or septic arthritis, both of which can be ruled out through knee joint aspiration and appropriate laboratory investigations.

X-ray remains the preferred imaging modality for the diagnosis. Radiographic findings may reveal varying quantities of irregularly shaped calcifications in a periarticular location, especially around the attachment of tendons or ligaments. Calcific deposits may present as cloudy or spotty during the resorptive or post-calcification stages. MRI imaging may demonstrate a low-signal abnormality and edematous changes around or within the affected ligament. It is essential to distinguish the radiological changes of calcification from osteophytes and bony avulsion fractures on MRI scans (19). The signal intensity on MRI is directly associated with the concentration of calcium within the lesion and may not consistently exhibit a hypointense signal. While a single MRI examination alone may not be sufficient for diagnosing calcific tendinitis without x-ray imaging, MRI can serve as a valuable surgical planning tool, offering superior visualization of the location and extent of the calcific deposit compared to x-ray imaging (Table 1) (20). Ultrasound is widely used for joint assessment and can detect calcifications, providing precise localization for shockwave therapy or arthroscopic treatment without exposing patients to radiation. x-ray radiographs demonstrate poor diagnostic sensitivity for detecting hydroxyapatite crystal deposits in the early disease stages. Whereas ultrasound can identify periarticular calcific deposits and visualize adjacent capsular and pericapsular hyperemia. It also offers an effective method to differentiate between symptomatic and asymptomatic calcifications. In asymptomatic calcific arthritis, power Doppler ultrasound hardly detects microvascularity within tendons or ligaments (21). In the present case, the x-ray and CT images displayed calcifications in close proximity to the bilateral femoral condyles. MRI findings indicated the presence of calcific deposits with hypointense signals affecting both the MCL and LCL.

**Table 1:** Summary of radiological features in the present case (x-rays, CT scan, and MRI).

Imaging modality	Characteristics
x-ray	Dense, well-defined nodular hyperdense opacities.
CT	Calcific deposits appear as high-density opacities with higher resolution than x-ray, enabling detailed visualization. 3D reconstruction assists in location.
MRI	Calcific deposits exhibit hypointense signals, while edematous changes in the affected tissues demonstrate hyperintense signals. This imaging characteristic aids in differentiating osteophytes or avulsion fractures and assists in delineating the spatial relationship with surrounding anatomical structures.

There is currently no established gold standard for the treatment (22). As calcific periarthritis or tendinitis is typically a self-limiting condition, conservative treatment is generally preferred (10). Treatment approaches of the knee often mirror those used for patients with calcific tendinitis of the shoulder. Various conservative therapies, such as NSAIDs, extracorporeal shock wave therapy, local anesthetic injections, and ultrasound-guided percutaneous irrigation (UGPI), have been employed to manage painful calcific tendinitis (23, 24). In most cases, conservative treatment leads to alleviation of severe pain.

The therapeutic mechanism of extracorporeal shock wave therapy is multifactorial and not fully elucidated. Moreover, the procedure is associated with significant pain, high costs, and inconsistent efficacy, limiting its widespread adoption (25). In the literatures, UGPI for calcific tendinitis of the rotator cuff yields superior clinical outcomes compared to corticosteroid injection or extracorporeal shockwave therapy (26). A staged approach has been proposed: (a) ultrasound-guided needle fragmentation and lavage of the calcification, followed by (b) intrabursal corticosteroid administration to suppress post-procedural inflammation (27). However, UGPI is not recommended for patients with calcific deposits smaller than 5 mm in diameter or those exhibiting firm consistency.

In this presented case, however, after 2 weeks of systematic conservative treatment (including pharmacologic analgesia and physical therapy), the patient showed no significant improvement in either pain symptoms or knee range of motion (<90°). Due to persistent functional limitations and pain severely affecting daily living, the patient lost all confidence in non-surgical management and actively requested surgical intervention for a definitive solution. Open surgery is more invasive and disruptive to surrounding structures; in contrast, arthroscopic procedures offer a less traumatic approach and can expedite postoperative rehabilitation. During arthroscopic intervention, lavage not only clears residual calcifications but also eliminates inflammatory mediators. Additionally, arthroscopic debridement enables management of intra-articular lesions, such as congested synovium, providing an advantage over open surgery. These procedures typically yield excellent outcomes with prompt relief of symptoms. Therefore, arthroscopic surgery is indicated for selected cases, as it allows not only the excision of calcific deposits but also the concurrent treatment of any associated intra-articular pathologies. However, this approach necessitates hospitalization, anesthesia/sedation, and a prolonged rehabilitation period following the invasive procedure.

The management of this case had several limitations. First, the diagnosis primarily relied on laboratory tests and imaging studies (x-ray, CT, MRI), but ultrasound—a convenient and radiation-free modality—was not utilized, precluding observation of sonographic features of calcific periarthritis. Second, due to the patient's strong preference for surgical intervention, conservative treatments such as UGPI were not attempted. Given these limitations, future studies will focus on enhancing the diagnostic utility of ultrasound in calcific periarthritis and exploring the efficacy of physical therapy and ultrasound-guided interventional procedures for calcific disorders.

In conclusion, simultaneous calcific periarthritis affecting both the LCL and MCL is a rare pathological entity. The clinical course of MCL calcifications demonstrates the potential for asymptomatic presentation from onset to resolution. Calcific periarthritis is typically a self-limiting condition that may remain asymptomatic unless accompanied by inflammation. The utilization of MRI and x-ray imaging plays a crucial role in the accurate diagnosis of this condition, with surgery offering favorable outcomes in cases where conservative treatments prove ineffective. When considering surgical intervention, the arthroscopic technique—characterized by reduced trauma and shortened postoperative rehabilitation—represents a superior choice to open incision procedures.

### Patient perspective

After the onset of the disease, the pain and limited mobility of my knee made it difficult for me to walk and perform daily household tasks, resulting in a great decline in the quality of life, and anxiety. This anxiety was exacerbated when conservative treatment failed and I could not wait to request surgery. After the surgery, the pain relief was immediate and obvious, and I quickly resumed the normal life.

## Data Availability

The original contributions presented in the study are included in the article/Supplementary Material, further inquiries can be directed to the corresponding author.

## References

[1] Siegal DS Wu JS Newman JS Del Cura JL Hochman MG. Calcific tendinitis: a pictorial review. Can Assoc Radiol J. (2009) 60(5):263–72. 10.1016/j.carj.2009.06.008 19931132

[2] Song K Dong J Zhang Y Chen B Wang F Zhao J Arthroscopic management of calcific tendonitis of the medial collateral ligament. Knee. (2013) 20(1):63–5. 10.1016/j.knee.2012.05.004 22682211

[3] White WJ Sarraf KM Schranz P. Acute calcific deposition in the lateral collateral ligament of the knee. J Knee Surg. (2013) 26(S 01):S116–9. 10.1055/s-0032-1324815 23288749

[4] Seil R Litzenburger H Kohn D Rupp S. Arthroscopic treatment of chronically painful calcifying tendinitis of the supraspinatus tendon. Arthroscopy. (2006) 22(5):521–7. 10.1016/j.arthro.2006.01.012 16651162

[5] Cholet C Guerini H Pessis E Drapé JL Campagna R. Ultrasound features of painful intraosseous migration of pectoralis major tendinous calcifications with follow-up. J Ultrasound. (2020) 23(3):411–7. 10.1007/s40477-019-00395-0 31228123 PMC7441124

[6] Vinanti GB Pavan D Rossato A Biz C. Atypical localizations of calcific deposits in the shoulder. Int J Surg Case Rep. (2015) 10:206–10. 10.1016/j.ijscr.2015.04.011 25884610 PMC4430114

[7] Delbello F Spinnato P Aparisi Gomez MP. Calcific tendinopathy atypically located outside the rotator cuff: a systematic review. Curr Med Imaging. (2024) 20(1):E100423215585. 10.2174/1573405620666230410091749 37038296

[8] Meister BR Michael SP Moyer RA Kelly JD Schneck CD. Anatomy and kinematics of the lateral collateral ligament of the knee. Am J Sports Med. (2000) 28(6):869–78. 10.1177/03635465000280061601 11101111

[9] Wu WT Onishi K Mezian K Naňka O Wang B Su DC Ultrasound imaging of the posterior lateral corner of the knee: a pictorial review of anatomy and pathologies. Insights Imaging. (2024) 15(1):39. 10.1186/s13244-024-01606-x 38334861 PMC10857999

[10] Kamawal Y Steinert AF Holzapfel BM Rudert M Barthel T. Case report-calcification of the medial collateral ligament of the knee with simultaneous calcifying tendinitis of the rotator cuff. BMC Musculoskelet Disord. (2016) 17:283. 10.1186/s12891-016-1147-z 27411380 PMC4944491

[11] Hackett L Millar NL Lam P Murrell GA. Are the symptoms of calcific tendinitis due to neoinnervation and/or neovascularization? J Bone Joint Surg Am. (2016) 98(3):186–92. 10.2106/JBJS.O.00417 26842408

[12] Tennent TD Goradia VK. Arthroscopic management of calcific tendinitis of the popliteus tendon. Arthroscopy. (2003) 19(4):E35. 10.1053/jars.2003.50124 12671610

[13] Harvie P Pollard TC Carr AJ. Calcific tendinitis: natural history and association with endocrine disorders. J Shoulder Elbow Surg. (2007) 16(2):169–73. 10.1016/j.jse.2006.06.007 17188907

[14] Uhthoff HK. Calcifying tendinitis, an active cell-mediated calcification. Virchows Arch A Pathol Anat Histol. (1975) 366:51–8. 10.1007/BF00438677 804757

[15] Uhthoff HK Loehr JW. Calcific tendinopathy of the rotator cuff: pathogenesis, diagnosis, and management. J Am Acad Orthop Surg. (1997) 5(4):183–91. 10.5435/00124635-199707000-00001 10797220

[16] Mansfield HL Trezies A. Calcific tendonitis of the medial collateral ligament. Emerg Med J. (2009) 26(7):543. 10.1136/emj.2008.067710 19546285

[17] Hughes PJ Bolton-Maggs B. Calcifying tendinitis. Curr Orthop. (2002) 16(5):389–94. 10.1054/cuor.2002.0259

[18] Tibrewal SB. Acute calcific tendinitis of the popliteus tendon–an unusual site and clinical syndrome. Ann R Coll Surg Engl. (2002) 84(5):338. 10.1308/003588402760452475 12398128 PMC2504146

[19] Schindler K O'Keefe P Bohn T Sundaram M. The case: your diagnosis? Calcific tendonitis of the fibular collateral ligament. Orthopedics. (2006) 29(4):282. 373–5. 10.3928/01477447-20060401-03 16628983

[20] Zubler C Mengiardi B Schmid MR Hodler J Jost B Pfirrmann CW. MR arthrography in calcific tendinitis of the shoulder: diagnostic performance and pitfalls. Eur Radiol. (2007) 17:1603–10. 10.1007/s00330-006-0428-6 17036154

[21] Le Goff B Berthelot JM Guillot P Glémarec J Maugars Y. Assessment of calcific tendonitis of rotator cuff by ultrasonography: comparison between symptomatic and asymptomatic shoulders. Joint Bone Spine. (2010) 77(3):258–63. 10.1016/j.jbspin.2010.01.012 20434387

[22] Spinnato P Ponti F D’Agostino V Miceli M Guerra E Marinelli A Ultrasound-guided percutaneous irrigation of calcific tendinopathy outside the rotator cuff: short-term evaluation. Skeletal Radiol. (2022) 51(10):2039–44. 10.1007/s00256-022-04035-3 35366095

[23] Louwerens JK Sierevelt IN Kramer ET Boonstra R van den Bekerom MP van Royen BJ Comparing ultrasound-guided needling combined with a subacromial corticosteroid injection versus high-energy extracorporeal shockwave therapy for calcific tendinitis of the rotator cuff: a randomized controlled trial. Arthroscopy. (2020) 36(7):1823–33. 10.1016/j.arthro.2020.02.027 32114063

[24] Suzuki K Potts A Anakwenze O Singh A. Calcific tendinitis of the rotator cuff: management options. J Am Acad Orthop Surg. (2014) 22(11):707–17. 10.5435/JAAOS-22-11-707 25344596

[25] Galletti L Ricci V Andreoli E Galletti S. Treatment of a calcific bursitis of the medial collateral ligament: a rare cause of painful knee. J Ultrasound. (2019) 22(4):471–6. 10.1007/s40477-018-0353-y 30811015 PMC6838279

[26] Vassalou EE Klontzas ME Plagou AP Karantanas AH. Ultrasound-guided percutaneous irrigation of calcific tendinopathy: redefining predictors of treatment outcome. Eur Radiol. (2021) 31:2634–43. 10.1007/s00330-020-07334-2 33040221

[27] Messina C Sconfienza LM. Ultrasound-guided percutaneous irrigation of calcific tendinopathy. Semin Musculoskelet Radiol. (2016) 20(5):409–13. 10.1055/s-0036-1594285; Thieme Medical Publishers. 28002862

