# Supplementary material for: Case Report: Arthroscopic treatment of calcific periarthritis of the knee in the lateral and medial collateral ligaments
Source: Front Surg. 2025 Jul 14;12:1466031. doi: 10.3389/fsurg.2025.1466031 (PMC12301296; doi:10.3389/fsurg.2025.1466031)
Supplement: Supplementary file 1 [file Table1.pdf]

Time	Episode of Care	Examination and Treatment
August 13, 2021	Onset of knee joint pain	Imaging (X-ray/CT/MRI) confirmed calcific periarthritis.
September 1, 2021	Unsuccessful nonoperative treatment	Arthroscopic surgery was performed
November 2021	First follow-up visit	Pain-free with full return to normal daily activities and work
October 2023	2-year postoperative follow-up	No signs of recurrence with dissolution of calcification in the MCL
